# Identification of M2 macrophage-related genes for establishing a prognostic model in pancreatic cancer: *FCGR3A* as key gene

**DOI:** 10.32604/or.2024.055286

**Published:** 2024-11-13

**Authors:** ZHEN WANG, JUN FU, SAISAI ZHU, HAODONG TANG, KUI SHI, JIHUA YANG, MENG WANG, MENGGE WU, DUNFENG QI

**Affiliations:** 1 Department of Hepatopancreatobiliary Surgery, XuZhou Central Hospital Affiliated to Medical School of Southeast University, Xuzhou, 221000, China; 2 Department of Surgery, School of Medicine, Southeast University, Nanjing, 210000, China; 3 Department of Oncology, Albert Einstein College of Medicine, Bronx, NY 10461, USA

**Keywords:** Pancreatic ductal adenocarcinoma (PDAC), M2 macrophages, Weighted gene co-expression network analysis (WGCNA), CIBERSORT, Immunization, Prognosis

## Abstract

**Background:**

Pancreatic ductal adenocarcinoma (PDAC) has a rich and complex tumor immune microenvironment (TIME). M2 macrophages are among the most extensively infiltrated immune cells in the TIME and are necessary for the growth and migration of cancers. However, the mechanisms and targets mediating M2 macrophage infiltration in pancreatic cancer remain elusive.

**Methods:**

The M2 macrophage infiltration score of patients was assessed using the xCell algorithm. Using weighted gene co-expression network analysis (WGCNA), module genes associated with M2 macrophages were identified, and a predictive model was designed. The variations in immunological cell patterns, cancer mutations, and enrichment pathways between the cohorts with the high- and low-risk were examined. Additionally, the expression of FCGR3A and RNASE2, as well as their association with M2 macrophages were evaluated using the HPA, TNMplot, and GEPIA2 databases and verified by tissue immunofluorescence staining. Moreover, *in vitro* cell experiments were conducted, where FCGR3A was knocked down in pancreatic cancer cells using siRNA to analyze its effects on M2 macrophage infiltration, tumor proliferation, and metastasis.

**Results:**

The prognosis of patients in high-risk and low-risk groups was successfully distinguished using a prognostic risk score model of M2 macrophage-related genes (*p* = 0.024). Between the high- and low-risk cohorts, there have been notable variations in immune cell infiltration patterns, tumor mutations, and biological functions. The risk score was linked to the manifestation of prevalent immunological checkpoints, immunological scores, and stroma values (all *p* < 0.05). *In vitro* experiments and tissue immunofluorescence staining revealed that FCGR3A can promote the infiltration or polarization of M2 macrophages and enhance tumor proliferation and migration.

**Conclusions:**

In this study, an M2 macrophage-related pancreatic cancer risk score model was established, and found that FCGR3A was correlated with tumor formation, metastasis, and M2 macrophage infiltration.

## Introduction

Pancreatic cancer, a solid tumor with a dismal diagnosis (5-year survival rate below 9%), consists primarily of pancreatic ductal adenocarcinoma (PDAC), representing 80% to 90% of cases [1,2]. By 2030, PDAC is anticipated to be the other most prevalent reason for tumor-related fatalities [3]. Initially, PDAC manifests slowly and lacks specific early diagnostic markers, which often leads to patients being diagnosed at an advanced stage with distant metastases, rendering them ineligible for curative surgery [4]. Therefore, the only available treatments are systemic drugs such as FOLFIRINOX (5-fluorouracil, folinic acid [leucovorin], irinotecan, and oxaliplatin), gemcitabine plus nab-paclitaxel, and radiotherapy such as stereotactic body radiation treatment (SBRT) [5]. Unfortunately, an extensive stroma serves as an external barrier to prevent traditional chemotherapy medications from penetrating PDAC [6]. Furthermore, a rich tumor immune microenvironment (TIME) including dendritic cells, macrophages, B-cells, T-cells, and a variety of immune-related cells or compounds has been linked to PDAC [7]. Tumor vaccines, CAR-T treatment, and immune checkpoint inhibitors (ICI) are among the immunotherapies that are ineffective against tumors because of their influence of TIME on the phenotypic variations and actions of the tumor cells [8]. However, the infiltrated immunological cells that have been the most extensively in the TIME are tumor-associated macrophages (TAMs), significantly constituting around 11% of the cellular makeup within PDAC tissues [9]. In PDAC, TAMs primarily manifest as M2-type macrophages and a lower rate of overall survival in pancreatic cancer sufferers is associated with increased M2 macrophage abundance [10,11]. Extensive clinical evidence underscores the intimate link between M2 macrophage infiltration levels and poor prognosis across a wide range of cancers, attributed to M2 macrophages’ ability to fuel tumor progression by stimulating angiogenesis [12]. Furthermore, TAMs actively engage in crosstalk with cancer cells, fueling tumor advancement and maintaining an immunosuppressive milieu conducive to tumor immune evasion [13]. For instance, Hu et al. demonstrated that ALOX5 stimulates macrophages associated with cancer to become M2 polarized, resulting in pancreatic cancer infiltration and metastasis [14]. Additionally, Zhang et al. further confirmed that M2 macrophages facilitate communication with tumor cells via their secreted exosomes, promoting malignant tumor progression [15].

As pivotal drivers of tumor progression and metastasis, M2 macrophages have emerged as promising targets for novel therapeutic interventions in cancer management [16,17]. However, it’s still unclear precisely which mechanisms lead to M2 macrophage polarization and invasion in pancreatic cancer [18]. Consequently, urgent efforts are warranted to identify reliable biomarkers for assessing M2 infiltration levels in pancreatic cancer, thus paving the way for innovative strategies in targeted adjuvant therapy development. In this study, we used the xCell method and WGCNA to evaluate the TCGA and GEO datasets to determine a candidate gene set that included 73 genes linked to M2 macrophages in pancreatic tumors. Subsequently, we merged TCGA and GEO pancreatic cancer cohorts using the “SVA” R package and established a potential rating model using genes associated with M2 macrophages. This risk score model effectively stratifies patients based on risk scores and predicts patient outcomes. Through public database analysis, tissue immunofluorescence, and *in vitro* cell experiments, we identified that FCGR3A is associated with M2 macrophage infiltration or polarization and contributes to pancreatic cancer proliferation and metastasis. Such findings contribute to our knowledge of the predictive significance of M2 macrophage infiltration in PDAC and offer new targets for cancer treatments that are customized to the tumor microenvironment.

## Materials and Methods

### Data collecting and processing

We thoroughly examined the Gene Expression Omnibus (GEO) dataset (https://www.ncbi.nlm.nih.gov/geo/, accessed on 12 September 2024) for gene activity profiles of PDAC individuals with survival information. Ultimately, GSE183795, comprising 105 normal samples and 139 cancer samples were determined for the current analysis. The Cancer Genome Atlas (TCGA), which can be accessed at https://cancergenome.nih.gov, accessed on 12 September 2024 provided transcriptome activation profiles, somatic variant data, and patient information associated with PDAC. Transcripts per million (TPM) formatted data on expression has been obtained from TCGA, encompassing 179 tumor specimens and 4 normal specimens. Tumor mutation burden was determined through the calculation of tumor-specific mutated genes. Subsequently, cases lacking follow-up data and normal samples were excluded, resulting in the inclusion of 178 cases for subsequent evaluation. Integrating the “SVA” R module and standardize the two databases (TCGA-PAAD and GSE183795). Eventually, we ran our analysis on 317 cancer specimens.

### Weighted gene co-expression network analysis

The ‘Xcell’ R package (version 1.1.0) used in pancreatic cancer cohorts from TCGA and GEO to determine the proportion of tumor-infiltrating immune cells (TIICs). Using the mean M2 macrophage value as phenotypic data, samples from all cohorts were categorized into subjects with high and low M2 macrophage infiltration [10]. Then, in the pancreatic tumour cohorts from TCGA and GEO, the gene-expression patterns with at least 25% variability were selected as input for weighted gene co-expression network analysis (WGCNA) employing the “WGCNA” R tool (version 1.72.1). First, a topological overlap matrix (TOM) was produced by converting an adjacency matrix into an estimate of network interaction. The soft threshold β was then calculated using the ‘sft$powerEstimate’ function, with a threshold level of 0.25 and a minimum module size of 50. Ultimately, key modules displaying the highest correlation with tumor traits were identified based on Pearson correlations between module eigengenes (MEs) and features.

### Identification of hub M2-macrophage associated genes and development of a prognostic signature

Initially, the Wayne diagram tool (http://bioinformatics.psb.ugent.be/webtools/Venn/, accessed on 12 September 2024) was used to intersect the module genes associated with M2 macrophages identified by WGCNA in two different cohorts to find genes linked to M2 macrophages. Subsequently, the “SVA” R package (version 3.42.0) was applied to merge and standardize the TCGA and GEO datasets, resulting in 317 tumor specimens for prognostic model construction. For each of the 317 PDAC groups, a univariate Cox regression evaluation was conducted, and genes with *p* < 0.05 have been selected for more research. Moreover, the potential genes have been further improved, and a prediction model was developed with the R package “glmnet” (version 4.1.7) and the LASSO regression method [19]. The formula used to calculate the probability value was risk score = (expression of Gene A × β1) + (expression of Gene B × β2) +... + (expression of Gene n × βn), where β is the coefficient of regression of the genes correlated with the signature.

### Validation of the prognostic signature

Using the previously developed risk estimation, a risk score was allocated to all of the 317 PDAC specimens. Using the average of the probability score as the cutoff criterion, samples were divided into low and high risk subdivisions. To evaluate prognostic differences, Kaplan-Meier (K-M) survival rates are initially generated using the R program “Survival” (version 3.5.0). The area under the curve (AUC) between the risk estimate, cancer stage, and tumor grade has been compared using receiver operating characteristic (ROC) curves to determine the therapeutic validity of the risk index model.

### Design and evaluation of nomograms

Using the “rms” R package (version 6.6.0), a predictive nomogram was produced including the probability value and clinical variables considering the phase and tumor level to forecast the overall survival (OS) rates of PDAC patients for the first, third, and five years [20]. The efficacy of the predictive nomogram was evaluated by comparing predicted and observed OS using calibrating plots. Furthermore, the “timeROC” R program (version 0.4.0) was implemented to perform 3- and 5-year ROC curve experiments to evaluate the nomogram’s quality.

### Protein-protein interaction (PPI) analysis

A PPI structure of those 73 genes linked to M2 macrophages have been identified by querying the STRING dataset (http://string-db.org/, accessed on 12 September 2024). The number of nodes inside the network was then computed. After that, the R package “igraph” (version 1.3.5) was adopted to locate and display the nodes in the centre.

### Functional annotation analysis

The ‘clusterProfiler’ R program (version 4.2.2) was employed to perform pathway enrichment analyses for Gene Ontology (GO) subjects correlated to the 73 M2 macrophage relevant genes. These expressions include biological processes (BP), cellular components (CC), and molecular functions (MF) [21]. A threshold of *p* < 0.05 was used for statistical significance for analyzing enrichment data.

### Evaluation of immune infiltration

The R program “CIBERSORT” (version 0.1.0) was used to evaluate the immunological cell fractions in each of the 317 PDAC samples that were obtained from the TCGA and GEO datasets. A total of 1000 aligned default signature matrices were used to enhance algorithm accuracy. The corresponding proportions of 22 TIICs and the related CIBERSORT *p*-values were gathered using Monte Carlo sampling. Only samples with a CIBERSORT *p*-value of below 0.05 were chosen for a further analysis [22].

### Gene set enrichment analysis (GSEA)

Using a GSEA, the gene expression levels of a predefined set of genes were compared between low and high-risk individuals to identify significant variations. We have selected the corresponding gene set “c2.cp.kegg.Hs.symbols.gmt.” To assess statistical significance, a modified *p* < 0.05 threshold was employed [23].

### Differential mutational profiles among various group

We investigated the mutational landscape of the top 20 genes most commonly mutated in PAAD samples. Furthermore, we used the R package ‘maftools’ (version 2.10.5) to perform a comparison study of the changes between both high and low risk populations.

### Relationships between risk score model and immunization status

In the 317 PDAC samples, we employed the ssGSEA method by ‘GSVA’ R package (version 1.42.0) to compute 29 immune-related functional analysis. The stromal score, immunological cell infiltration score, and purity of tumor in the 317 PDAC samples were also determined using the ESTIMATE approach, which served to estimate the stromal and immunological cells within cancer cells. The ‘pheatmap’ R program (version 1.0.12) was then utilized to produce the heatmap. Finally, for assessing the immunological checkpoint expressions (CD274 and CTLA4) across multiple groups, the R program ‘corrplot’ (version 0.92.0) has been utilized.

### Gene expression associated with the risk score model

The Human Protein Atlas (HPA) Database (https://www.proteinatlas.org/, accessed on 12 September 2024) and TNMplot Data (https://tnmplot.com/analysis/, accessed on 12 September 2024) have been employed to compare the protein and transcript of FCGR3A and RNASE2 expression levels between pancreatic tumor tissue and nearby healthy tissue. Moreover, M2 macrophage-specific markers (CD163, CD206) have been examined for their associations with FCGR3A and RNASE2 by executing the GEPIA2 dataset (http://gepia2.cancer-pku.cn/#inde, accessed on 12 September 2024).

### Immunofluorescence staining

From patients with pancreatic cancer patients had radical pancreatic tumor resection at XuZhou Center Hospital, four formalin-fixed paraffin-embedded (FFPE) blocks of tissues have been identified. Individuals who had not received preoperative radiotherapy or chemotherapy and had a postoperative pathological examination that confirmed the diagnosis of PDAC were included. Tissue blocks were sectioned using a rotary microtome (REWARD, S710, Shenzhen, China) to obtain 4 µm thick tissue slices. Immunofluorescence staining was conducted by Servicebio, Wuhan, China. The staining protocol adhered to the company’s instructions, detailed in a previous publication [24]. For immunofluorescence staining of FCGR3A, RNASE2 and CD163, FCGR3A was stained with green, RNASE2 was labeled with yellow and CD163 was labeled with red. Cell nuclei were counterstained with 4,6-diamidino-2-phenylindole (DAPI) (blue) (Servicebio, G1012, Wuhan, China). Antibodies used in the immunofluorescence panels included RNASE2 (Incubation concentration: 1:500) (Abcam, ab238562, Cambridge, UK), FCGR3A (Incubation concentration:1:500) (Abcam, ab287421, Cambridge, UK), and CD163 (Incubation concentration: 1:1000) (Servicebio, GB15340-100, Wuhan, China), these primary antibodies were allowed to incubate overnight. Images were captured using a scanner, and image data were processed using CaseViewer 2.4 software (3DHISTECH, Hungary).

### Cell culture

The cell repository of the Chinese Academy of Sciences in Shanghai, China from which the human pancreatic tumour cell lines PANC-1, CAPAN-2, and BXPC-3, as well as the pancreatic ductal cells HPNE and the monocytic cell line THP-1, were obtained. With the assistance of LDT Bioscience (Shanghai, China), after being examined for mycoplasma contamination, each cell was proven to be mycoplasma-free, and they were authenticated using STR profiling. The THP-1 cells were cultured in 1640 media (Gibco, Invitrogen, Cat#11875093, Grand Island, USA), supplemented with 10% fetal bovine serum (Gibco, Invitrogen, Cat#10099141C, Grand Island, NE, USA) and 1% penicillin-streptomycin (Beyotime, Cat#C0222, Shanghai, China). The additional lines of cells were grown in DMEM (Gibco, Invitrogen, Cat#11965092, Grand Island, NE, USA), supplemented with 10% fetal bovine serum (Gibco, Invitrogen, Cat#10099141C, Grand Island, NE, USA) and 1% penicillin-streptomycin (Beyotime, Cat#C0222, Shanghai, China), and cultured at 37°C in a moist environment with 5% CO_2_. To encourage the development of macrophages, THP-1 cells were cultured for two days at 37°C in a cell incubator using 100 ng/mL phorbol 12-myristate 13-acetate (PMA; Abcam, UK).

### Analysis of quantitative real-time PCR (qRT-PCR)

Using the Trizol reagent (Invitrogen, Cat#15596026CN, Carlsbad, CA, USA) per the manufacturer’s instructions, the entire RNA has been extracted from the cells. A NanoDrop spectrophotometer (Thermo Fisher Scientific, ND-3300, Franklin, MA, USA) was used to measure the concentration of RNA. Then, using the SweScript RT I First Strand cDNA Synthesis Kit (Servicebio, Cat#G3330-50, Wuhan, China), 1 μg of total RNA was reverse-transcribed into cDNA. After that, 2× SYBR Green qPCR Master Mix (Servicebio, Cat#G3326-01, Wuhan, China) was used to perform qRT-PCR on the resultant cDNA. The 2^−ΔΔCt^ approach was employed to analyse the extent of gene expression. Table A1 contains a list of primer sequences.

### Western blot

The BCA Protein Assay Kit (Beyotime, Cat#P0009, Shanghai, China) has been employed to assess the concentrations of all protein samples following they were separated using RIPA lysis buffer (Servicebio, Cat#G2002-100ML, Wuhan, China). Protein samples (30 μg per well) were resolved using sodium dodecyl sulfate-polyacrylamide gel electrophoresis (SDS-PAGE) (Epizyme, Cat#PG112, Shanghai, China), with protein markers in the first and last wells to indicate molecular weight. Electrophoresis was performed by starting with a voltage of 80 V. Once the protein markers reached the separating gel, voltage was increased to 120 V and continued until the blue dye front reached the bottom of the gel. Then, using a constant current of 250 mA, wet transfer technique was performed for approximately 1 h to transfer the proteins onto polyvinylidene fluoride (PVDF) membranes (Millipore, Burlington, VT, USA). The PVDF membranes were then blocked for two hours with 5% skim milk and treated with the primary antibodies (FCGR3A (Abcam, ab287421, Cambridge, UK); GAPDH (Proteintech, 60004-1-Ig, Wuhan, China)). Following the primary antibody incubation, incubate the secondary antibody specific to the species of the primary antibody for 1 hour at 4. Signal detection and visualization were performed using BeyoECL Star (Beyotime, Cat#P0018AM, Shanghai, China).

### Small interfering RNA (siRNA)

GenePharma Co., located in Shanghai, China, designed and manufactured siRNA that targets FCGR3A (refer to Table A2). By utilizing siRNA-mate (GenePharma, Shanghai, China) and as per company’s instructions, pancreatic tumor cells were transfected with these siRNAs. The procedure involves seeding cells in a 24-well plate, allowing them to reach the stage of logarithmic growth, and then preparing the transfection complex by combining 2 µL of siRNA-mate transfection reagent with 10 pmol of siRNA in 100 µL of OPTI-MEM (Gibco, Cat#31985070, Grand Island, NE, USA). Next, each well in the 24-well plate received 100 µL of the transfected complex, increasing the total dose of siRNA to 16.7 nM. After that, the cells had been cultured at 37°C. A new medium had taken the place of the previous ones. The cells were employed in additional studies after 48 h.

### Flow cytometry

After being resuspended in ice-cold 1 × PBS, cells were treated for 30 min at 4°C in the dark with CD68 and CD163. After that, the cells were cleaned with 1 × PBS and spun for five minutes at 1000 rpm at 4°C using a high-speed low-temperature centrifuge (Servicebio, SLX-1024F, Wuhan, China). This was followed by a half-hour incubation at 4°C in the dark with a second antibody that had FITC labeling. Following incubation, the cells underwent one more PBS wash before being examined with a Thermo Fisher Scientific, Franklin, MA, USA, flow cytometer.

### Cell proliferation assay

For the Cell Counting Kit-8 (CCK-8) assay, 1000 cells were seeded into each well of a 96-well plate. Subsequently, 100 µL of fresh DMEM media and 10 µL of the solution of CCK-8 (Beyotime, Cat#C0038, Shanghai, China) went in to each well, and the resulting mixture was incubated for two hours at 37°C. A BioTek ELx800 Absorbance Microplate Reader was used to quantify each well’s absorbance at 450 nm. The 5-Ethynyl-2′-Deoxyuridine (EdU) test was conducted using 96-well plates with cells seeded at a density of 1 × 10^4^ per well. Then, Beyotime, China’s BeyoClickTM EDU-488 Cell Proliferation Detection Kit was used. After being cultivated, the cells were fixed, washed, labeled with EdU, and stained with nuclear dye. The number and intensity of green fluorescence were finally studied with a fluorescence microscope (Shanghai Yuehe, YH-YHF40, Shanghai, China).

### Migration and invasion assay

Wound Healing Assay: In 6-well plates, cells (5 × 10^4^ cells per well) were developed until they achieved 80%–90% confluence. To produce a straight scratch, a 200 µL plastic pipette tip was employed. The wound edges were marked and photographed at the start and 48 h later with ImageJ software (version 8.0) (NIH, Bethesda, MD, USA). Cell migration distance was quantified, and wound closure percentage was calculated. Transwell Migration and Invasion Assay: In the leading chambers of Transwell plates (24 wells, 8 μm pore size; Falcon), 4 × 10^4^ cells were introduced in 200 µL of serum-free media for the migration experiment. A volume of 400 µL of DMEM containing 10% FBS was added to the bottom chambers. The plates were next incubated for two days at 37°C. The cells were incubated for 15 min, after they were fixed with 4% formaldehyde and stained for 10 min with 0.1% crystal violet (CV). Following the removal of the cells from the membrane’s upper side, a microscope (Nikon, Eclipse E200 POL, Tokyo, Japan) was used to examine the migrating cells on the lower surface. The identical process was used for the invasion assay, with the exception that Matrigel matrix (Corning, Cat#354277, New York, NY, USA) was pre-coated onto the Transwell inserts.

### Statistical analysis

To determine the differences between the two groups, the *t*-test was applied. Pearson’s analysis was employed for calculating correlation coefficients. For comparing overall survival disparities among various groups, the *t*-test was employed to determine the variations between the two distinct groups and Log-rank test was applied. The threshold for significance of below 0.05 was selected. Using the R program, a statistical examination was performed. All experiments were independently repeated three times.

## Results

### Identification of M2 macrophage-related genes using WGCNA

**Figure 1:**
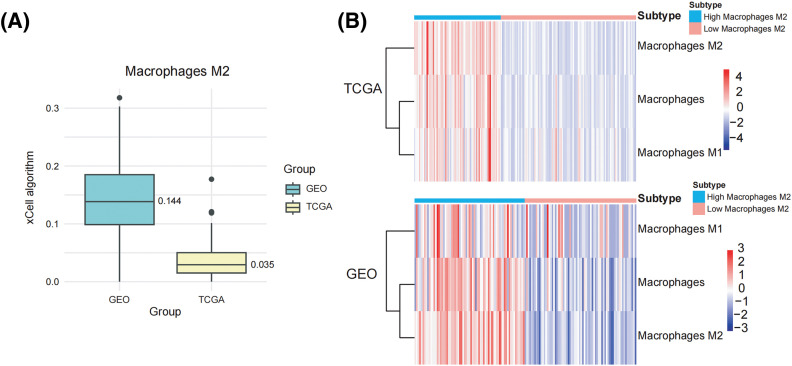

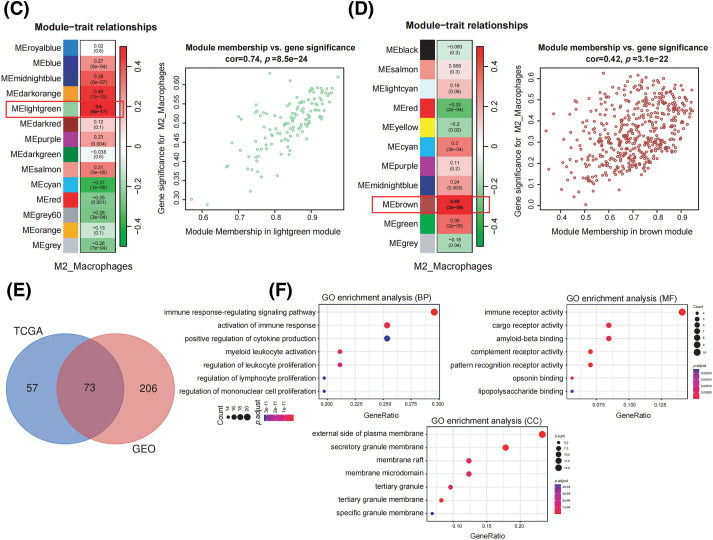
Identification of M2 macrophage-related genes using WGCNA. (A) M2 macrophage proportions were obtained from pancreatic cancer cohorts in TCGA and GEO databases using the Xcell algorithm. (B) Heatmap illustrating M2 macrophage proportions. (C) Heatmap showing the association between M2 macrophages and module eigengenes (MEs) in the TCGA Group. (D) Heatmap association between MEs and M2 macrophages in the GEO Cohort. (E) Venn plot of light green module genes (genes in the TCGA tumor cohort that show a significant association with M2 macrophages) and brown module genes (The GEO pancreatic carcinoma cohort’s genes exhibit a strong positive correlation with M2 macrophages). (F) Functional annotation of 73 M2 macrophage-related genes.

**Figure 2:**
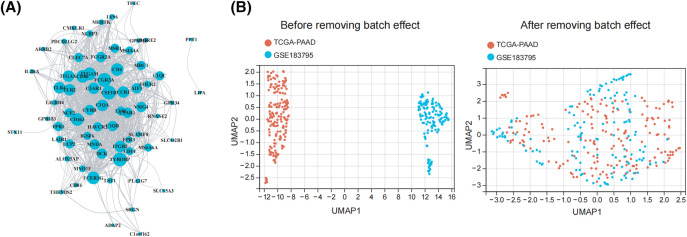

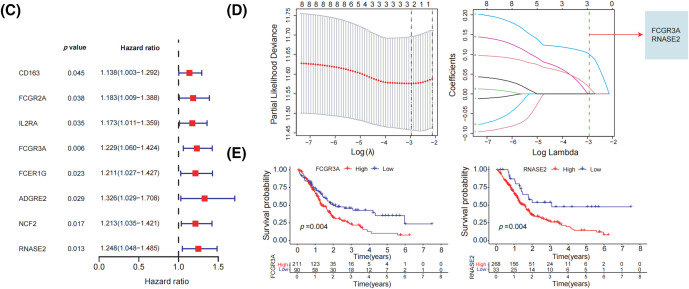
Identification of prognostic characteristics related to M2 macrophage. (A) The network of the genes in 73 M2 macrophage-related genes. (B) UMAP plot of TCGA and GEO datasets pre- and post-normalization. (C) When 73 M2 macrophage-related genes were subjected to a univariate Cox regression study, it showed that 8 genes had a strong association with the survival of patients with pancreatic tumors. (D) The optimal parameters (lambda, λ) were screened by ten-fold cross-validation, and the OS-related genes were subjected to LASSO regression. (E) Kaplan-Meier evaluation of FCGR3A and RNASE2 expression groups with high or low activity.

**Figure A1:**
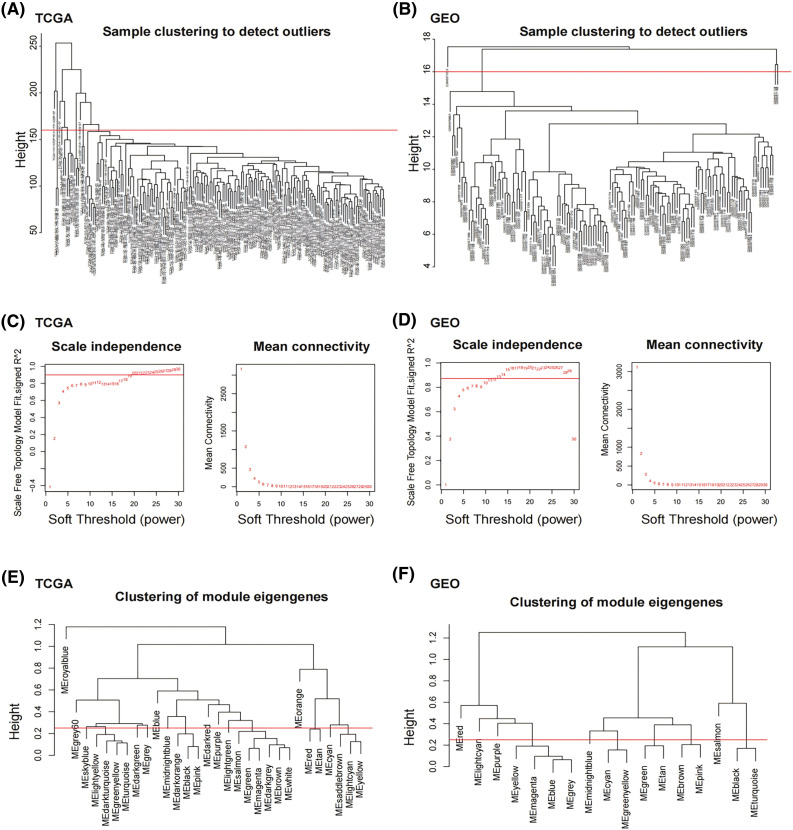
M2 macrophage-related modules identified by WGCNA. (A) Sample clustering analysis was conducted in TCGA dataset. (B) Sample clustering analysis was conducted in GEO dataset. (C) Network topology analysis for various soft-thresholding powers in TCGA dataset. (D) Network topology analysis for various soft-thresholding powers in GEO dataset. (E) Clustering of MEs in TCGA dataset. (F) Clustering of MEs in GEO dataset. The red line indicates cut height (0.25).

We collected 179 PDAC samples from the TCGA repository and 139 PDAC samples from the database of GEO. First, we used the Xcell approach to determine the ratio of M2 macrophages for all data sets individually. Then, PDAC samples were categorized into both high and lower M2 macrophage infiltration sets based on the median M2 macrophage proportion score (Fig. 1A,B). To recognize M2 macrophage-associated hub genes, we performed WGCNA. Firstly, sample clustering analysis was conducted using the ‘Hclust’ function, with a height of 160 for the TCGA dataset and 16 for the GEO dataset (Fig. A1A,B). The soft threshold was determined and set to 19 for TCGA and 12 for GEO using ‘sft$powerEstimate’ (Fig. A1C,D). Subsequently, after TOM network construction with a height of 0.25 (Fig. A1E,F), the light green module (containing 130 genes) in TCGA and the brown module (containing 279 genes) in GEO were identified to have the highest significant positive correlation between module eigengenes (MEs) and features (Fig. 1C,D). After intersecting these genes, 73 M2 macrophage-related genes were retained (Fig. 1E). In addition, an analysis of functional enrichment was carried out to understand the roles of such genes, identifying pathways associated with immunity (*e.g*., regulation of mononuclear cell proliferation, immune response-regulating signaling pathway, and positive regulation of cytokine production, etc.) (Fig. 1F). Lastly, STRING was performed to establish the PPI network of the aforementioned gene and genes with a threshold weight > 0.4 were depicted in Fig. 2A.

### Identification of prognostic characteristics related to M2 macrophages

Initially, we integrated the TCGA and GEO datasets after successfully removing batch effects. The UMAP plot revealed that after batch effect removal, samples from both datasets were intricately clustered together, indicating that batch effects were effectively eliminated (Fig. 2B). The 73 M2 macrophage-related genes were then evaluated by univariate Cox regression analysis, which showed that 8 genes had significant correlations with the survival rate of PDAC patients (*p* < 0.05) (Fig. 2C). Following this, to determine which gene prognosis was most optimal, LASSO analysis was conducted (Fig. 2D). Therefore, two genes were found to be significant: RNASE2 and FCGR3A. The Kaplan-Meier analysis showed that patients with a low level of RNASE2 and FCGR3A had a longer overall survival (OS) than those with elevated levels of both genes (Fig. 2E).

### Formation of predictive signature in pancreatic cancer

**Figure 3:**
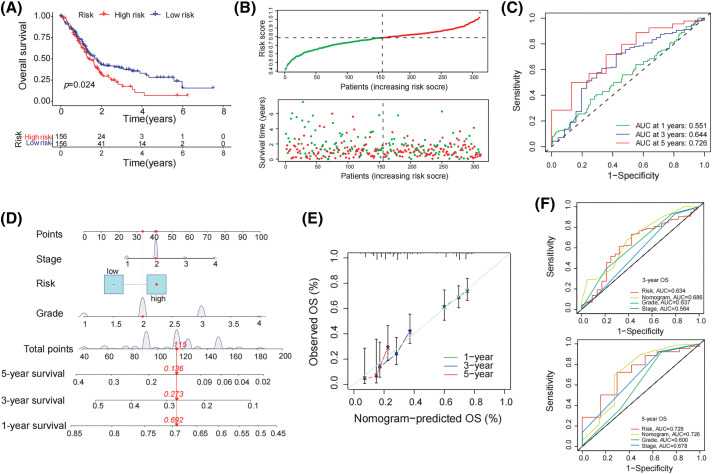
Formation of predictive signature in pancreatic cancer. (A) Kaplan-Meier survival curve demonstrates significantly lower overall survival in the high-risk group compared to the low-risk group. (B) The mean survival time of those in the high-risk group was significantly shorter than that of patients in the low-risk group, considering the distribution of risk evaluations and the survival status of pancreatic tumor patients. (C) The 1-year, 3-year, and 5-year survival rates of patients with pancreatic carcinoma were determined by the AUC of the ROC curve to determine highly predictive certain prognostic traits (D) Establish a nomogram including tumor stage, risk, and tumor grade to predict 1-year, 3-year, and 5-year overall survival of PDAC patient (E) The nomogram’s calibration curve for the overall survival following one, three, and five years (F) In the ROC study for 3-year and 5-year survival rates, the predictive value of the risk index model, tumor level, tumor stage, and all of these parameters was assessed.

To establish a prognostic signature, FCGR3A and RNASE2 were used. The risk index was computed as follows: Risk Score is equal to (0.103 × FCGR3A expression) + (0.019 × RNASE2 expression). Based on the median risk score, patients were divided into both high- and low-risk groups. The high-risk category showed a significantly reduced survival rate than the low-risk group, under the Kaplan-Meier survival study (Fig. 3A). Furthermore, overall survival duration was significantly less in the high-risk group than in the low-risk group, given the distribution of survival rate and risk evaluations (Fig. 3B). The degree of risk model’s specificity and sensitivity have been assessed using time-varying receiver operating characteristic (ROC) analysis, and the AUC has been determined (Fig. 3C). Moreover, using Stage, Grade, and risk score, a nomogram was designed to predict overall survival (Fig. 3D). The nomograms showed probability and the actual proportion of 1-, 3-, and 5-year overall survival showed remarkable consistency in the calibration curves (Fig. 3E). Finally, ROC curve findings showed that the probability index had more predictive ability than other clinically significant variables (3-year os: AUC = 0.634; 5-year os: AUC = 0.729) (Fig. 3F).

### GSEA and tumor mutations analysis for low and high-risk cohorts

**Figure 4:**
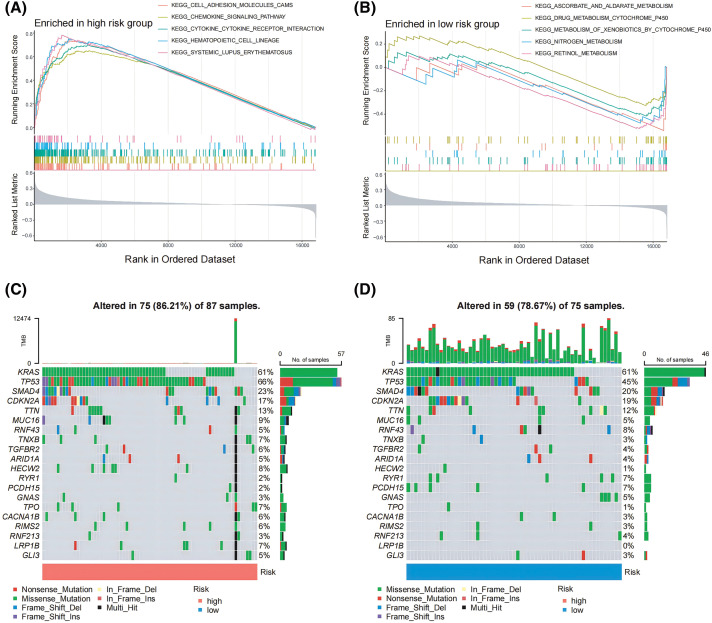
GSEA and tumor mutations analysis for lower and higher risk cohorts. (A, B) GSEA served to examine the pathways in a pancreatic cancer cohort that were strongly related to both low- and high-risk categories. (C, D) The top 20 frequently mutated genes in PAAD samples from multiple risk categories are shown in a cascade map that displays their mutant landscape.

To assess the enrichment of various gene families between the susceptible and lower-risk groups, we performed a GSEA study. KEGG_CELL_ADHESION_MOLECULES_CAMS, KEGG_CHEMOKINE_SIGNALING_PATHWAY, and KEGG_CYTOKINE_CYTOKINE_RECEPTOR_INTERACTION were the main gene categories that had an increase in the high-risk cohort (Fig. 4A). Similarly, KEGG_ASCORBATE_AND_ALDARATE_METABOLISM, KEGG_DRUG_METABOLISM_CYTOCHROME_P450, and KEGG_METABOLISM_OF_XENOBIOTICS_BY_CYTOCHROME_P450 had been linked to the set of genes that were predominant in those with low risks (Fig. 4B). Afterward, an analysis was conducted to compare the differential mutation profiles among various risk groups. Initially, it was shown that the group with a higher risk had a much more frequent rate of tumor mutations (86.21% *vs*. 78.67%) compared to the low-risk group. A distinct pattern was found upon further analysis of the top 20 modified genes. PDAC-related mutations, including TP53 (66%) were found more frequently in the group with higher risk, but KRAS (61%) mutations did not differ statistically between the low- and high-risk groups (Fig. 4C,D).

### Patterns of TIICs between high- and low-risk groups

**Figure 5:**
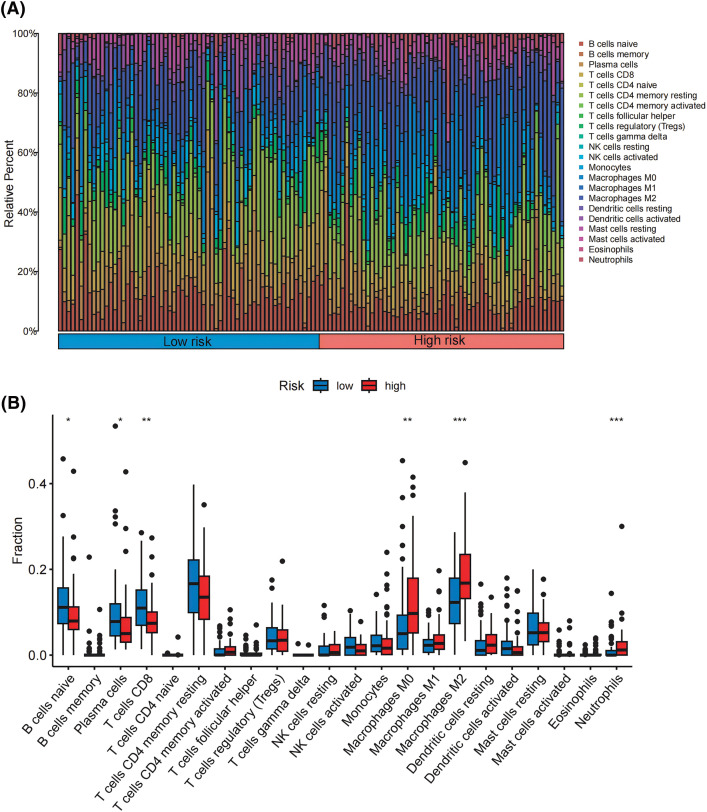
Patterns of TIICs between high- and low-risk groups. (A) Analysis of the infiltration patterns of 22 immune cells in 288 pancreatic cancer samples. (B) Comparison of the infiltration proportions of 22 immune cells between the high-risk and low-risk groups. *p < 0.05, **p < 0.01, ***p < 0.001.

Following the criteria of CIBERSORT with *p* < 0.05, we included 288 pancreatic cancer samples for subsequent analysis of infiltrated immunological cells, depicting the landscape of TIICs in PDAC, as illustrated in Fig. 5A. Subsequently, the TIIC variations between the high- and low-risk groups have been compared. Remarkably, we observed that the high-risk group had fewer B cells and CD8+ T cells than the low-risk group. On the other hand, the highly susceptible group’s levels of M0 macrophages, M2 macrophages, and neutrophils were notably higher (Fig. 5B), indicating patients in that group might prevent immune surveillance, which may result in developing tumors and poor prognosis.

### Relationships between the risk score model and immunization status

**Figure 6:**
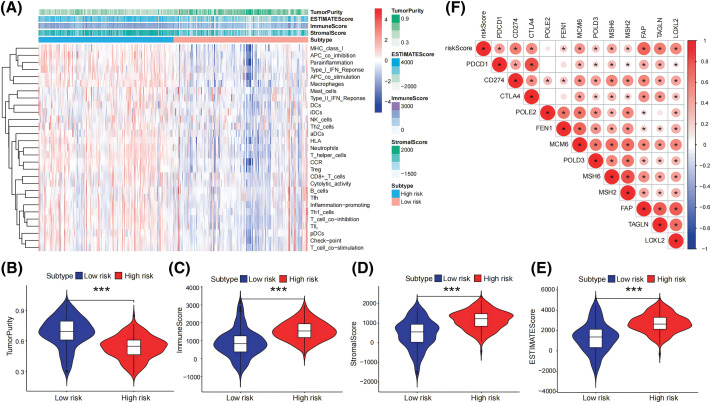
Relationships between risk score model and immunization status. (A) The levels of expression of 29 immune-related gene functions were described using ssGSEA and the ESTIMATE algorithm, and an analysis of ESTIMATE scores between high- and low-risk categories was done. (B–E) In the pancreatic tumor cohort, the stromal, immune, and ESTIMATE scores were obtained and compared between those with high and low risk employing the ESTIMATE program (F) A heatmap that compares the activity of immune checkpoint molecules across groups at both low and high risk. *p < 0.05, ***p < 0.001.

We also used the ESTIMATE method for assessing the immunological and stromal scores between the low- and high-risk groups. The group with a significant risk had considerably reduced tumor purity (all *p* < 0.05) and significantly higher stromal and immunological scores as compared to the minimal-risk group (Fig. 6A–E). Subsequently, based on 29 immune-related functional analysis parameters, a heatmap demonstrated significant variances between the higher and lower risk cohorts, comprising MHC_class_I, APC_co_inhibition, Parainflammation, Type_I_IFN_Reponse, APC_co_stimulation, Macrophages, Mast_cells, Type_II_IFN_Reponse, DCs, iDCs, NK_cells, Th2_cells, etc. (Fig. 6A). Finally, immune checkpoint expression patterns varied between the two cohorts. The heatmap that displayed the expression rates between the groups with the highest and lowest risk showed substantial variations in the levels of PDCD1, CD274, CTLA4, FEN1, MCM6, POLD3, MSH6, MSH2, FAP, TAGLN, and LOXL2 (all *p* < 0.05; Fig. 6F).

### M2 Infiltration in pancreatic tumors corresponds to high expression of FCGR3A

**Figure 7:**
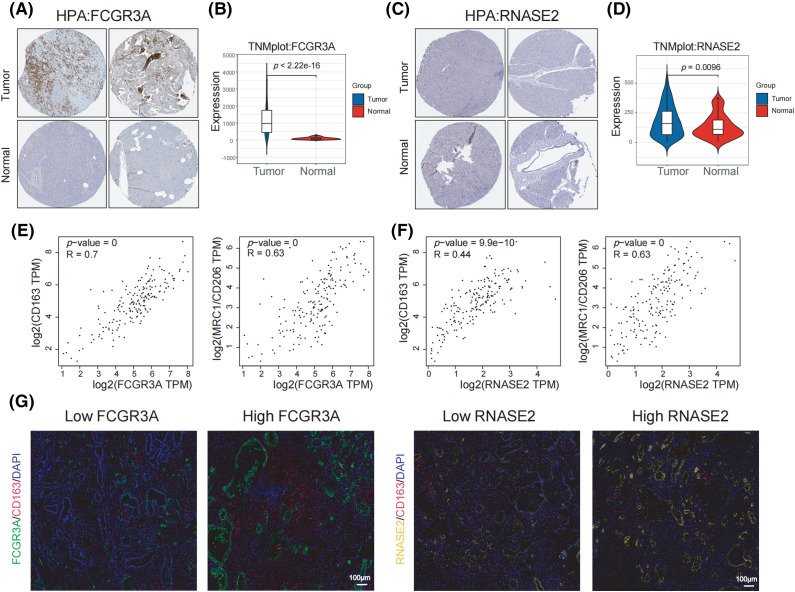
M2 infiltration in pancreatic tumors corresponds to high expression of FCGR3A. (A, B) The transcript abundance and protein expression levels of FCGR3A in tissues taken from the TNMplot and HPA datasets. (C, D) The RNASE2 protein and mRNA levels of expression in tissues were selected from the HPA and TNMplot datasets. (E) Using the GEPIA2 database, the link between FCGR3A expression and M2 macrophages (CD163 and CD206) in pancreatic tumor tissues was studied. (F) The correlation between RNASE2 expression and M2 macrophages (CD163 and CD206) in pancreatic cancer tissues was analyzed using the GEPIA2 database. (G) Immunofluorescence staining was used to analyze the correlation between the expression of FCGR3A and RNASE2 and M2 macrophage infiltration in PDAC.

Through a study of the HPA repository, it was possible to determine that cancerous pancreatic tissues had higher levels of FCGR3A protein expression than normal tissues executed (Fig. 7A), but there was not a significant distinction in RNASE2 protein expression between cancer and healthy tissues (Fig. 7C). Additionally, TNMplot database analysis revealed that tumor tissues had greater levels of FCGR3A and RNASE2 mRNA expression than healthy tissues (Fig. 7B,D). Further investigation using the GEPIA2 database showed that the expression levels of FCGR3A and RNASE2 were positively correlated with RNASE2 and M2 macrophage markers (CD163 and CD206) (Fig. 7E,F). Furthermore, we discovered that samples exhibiting elevated FCGR3A expression had a higher level of CD163 activity than those with low FCGR3A expression, based on FFPE sections (Fig. 7G). On the other hand, samples with both elevated and decreased RNASE2 expression did not differ in their production of CD163 (Fig. 7G). These results imply that M2 macrophage infiltration and FCGR3A expression in pancreatic cancer tissues are linked, which might explain that PDAC patients have an unfavorable prognosis.

#### In vitro experiments confirm FCGR3A promotes M2 polarization and facilitates pancreatic cancer proliferation and metastasis

**Figure 8:**
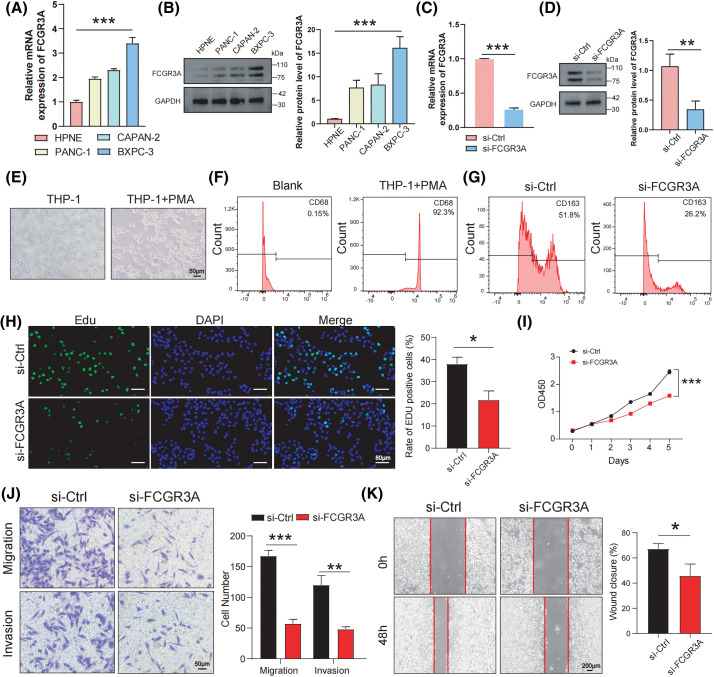
*In vitro* experiments confirm FCGR3A promotes M2 polarization and facilitates pancreatic cancer proliferation and metastasis. (A, B) By using western blot and qRT-PCR, the expression levels of FCGR3A in HPNE and pancreatic tumor cells were examined. The control gene is GAPDH (C, D) qRT-PCR and western blot validation of transient knockdown of FCGR3A in BXPC-3. GAPDH is the control gene. (E) THP-1 cells were induced by PMA *in vitro* to obtain M0 macrophages. (F) Flow cytometry showed a significant increase in CD68 expression in the THP-1+PMA group, indicating that THP-1 cells were successfully induced into M0 macrophages. (G) In the indirect co-culture of BXPC-3 cells and M0 macrophages, knocking down FCGR3A in BXPC-3 cells led to a decrease in the M2 polarization of macrophages. (H, I) BXPC-3 cells infected with si-Ctrl or si-FCGR3A were harvested for Edu assay and CCK-8 assay. (J, K) The transwell and wound healing assays demonstrated that knocking down FCGR3A in BXPC-3 cells significantly reduced the migration and invasion abilities of pancreatic cancer cells. *p < 0.05, **p < 0.01, ***p < 0.001.

A variety of *in vitro* studies were carried out to examine the involvement of FCGR3A in the evolution of PDAC. When compared to normal pancreatic ductal epithelial cells (HPNE), we first observed that pancreatic tumor lineages (PANC-1, CAPAN-2, and BXPC-3) had significantly higher expression levels of FCGR3A (Fig. 8A,B). We selected BXPC-3 cells, which exhibit high FCGR3A expression, and knocked down FCGR3A using si-RNA (Fig. 8C,D). Next, we induced macrophages from PMA-treated THP-1 cells and confirmed macrophage differentiation using flow cytometry for CD68 (Fig. 8E,F). Macrophages and BXPC-3 cells co-cultivated, and the results showed that BXPC-3 cell knockdown of FCGR3A decreased macrophage M2 polarization (Fig. 8G). Moreover, it was determined by the EdU and CCK-8 assays that inhibiting FCGR3A markedly decreased the ability of tumor cells in the pancreas to proliferate (Fig. 8H,I). Furthermore, FCGR3A knockdown significantly lowered the potential of pancreatic tumor cells to spread, as shown by transwell and wound healing studies (Fig. 8J,K).

## Discussion

Recent advances in molecular biology and high-throughput RNA sequencing have highlighted the critical role of the tumor microenvironment in cancer progression and metastasis [25–28]. In pancreatic ductal adenocarcinoma (PDAC), the highly immunosuppressive microenvironment and dense extracellular matrix suggest potential benefits of immunotherapy [29]. Macrophages in the tumor microenvironment can differentiate into M1 or M2 types [16]. M1 macrophages, activated by Th1 cytokines, aid in tumor cell killing, while M2 macrophages, activated by Th2 cytokines, exhibit immunosuppressive effects [30]. In PDAC, M2 macrophages are abundant and associated with poor prognosis, promoting tumor metastasis and inhibiting NK cell activity [31–33]. Understanding the mechanisms of M2 macrophage infiltration in PDAC could reveal new treatment targets and improve therapeutic strategies. Our study offers novel insights into the role of M2 macrophages and specific molecular targets in PDAC. A key finding is the identification of FCGR3A as a significant player in M2 macrophage polarization and its association with poor prognosis in PDAC. The relevance of FCGR3A in PDAC complements existing evidence on the role of the tumor microenvironment in cancer progression. While previous studies have implicated various immune cell types and molecular markers in PDAC, our research is among the first to specifically link FCGR3A with M2 macrophage activity [10,14,15]. This highlights a new potential therapeutic target that could be harnessed to mitigate the aggressive nature of PDAC.

As a transmembrane receptor mostly present on the surface of natural killer cells, FCGR3A (also known as CD16a) is a member of the FCGRs family and is essential for mediating immunological cell-based death of target tissues [34]. Li et al. discovered widespread upregulation of FCGR3A in various cancers [35]. According to survival analysis, FCGR3A predominates in most cancers and serves as a prognostic factor. Additionally, FCGR3A expression has also been linked to the infiltration of various immune cells, the expression of multiple immune checkpoint genes, and the repair of DNA mismatches in systemic cancers [35]. However, the prognostic significance of FCGR3A expression in pancreatic cancer and its correlation with immune infiltration remains unclear. The present study revealed that FCGR3A is involved in constructing the risk score cancer model, and effectively predicts patient prognosis, correlating with lower infiltration of B cells naive and CD8+ T cells, and higher infiltration of M2 Macrophages and Neutrophils. The association between FCGR3A and M2 macrophage infiltration found in PDAC tissues stained with immunofluorescence suggests that FCGR3A may play a role in promoting M2 macrophage infiltration, which results in decreased survival for PDAC individuals.

One of the 4 primary protein secretions released following eosinophil activation is RANSE2, also known as Ribonuclease A Family Member 2 (RNase2), or simply eosinophil-derived neurotoxic. It is a member of the RNaseA superfamily [36]. It was discovered that RNASE2 controls immunological responses by adjusting lymphocyte and macrophage activation. The regulation of that gene is linked to the stimulation of M2 macrophages, which produce substances that suppress the immune system and hinder the formation of tumors and immunological responses against them [37]. These findings are consistent with the GEPIA2 database, which shows an extensive positive association between RNASE2 and the M2 macrophage markers CD163 and CD206. Regrettably, in the immunofluorescence staining experiments of PDAC tissues, a correlation between RNASE2 and M2 macrophages was not observed. However, it was determined that FCGR3A was involved in M2 macrophage infiltration. Furthermore, cell experiments confirmed that FCGR3A is related to M2 macrophage polarization, as well as pancreatic cancer proliferation and development. Thus, FCGR3A could serve as a potential therapeutic target in pancreatic cancer. However, our study has limitations. Primarily, the research relies on *in vitro* models and publicly available datasets, which may not fully capture the complexity of *in vivo* conditions or account for patient heterogeneity. The immunofluorescence staining for RNASE2 did not reveal a clear correlation with M2 macrophages, indicating that further exploration is needed to validate its role in PDAC. Future research should focus on validating FCGR3A’s role *in vivo* assays to confirm its utility as a therapeutic target and investigating the precise mechanisms of FCGR3A in M2 macrophage infiltration and its impact on tumor progression will be crucial for translating these findings into clinical practice.

## Conclusion

In summary, we identified genes associated with M2 macrophages to establish a risk evaluation model for patients with pancreatic cancer. In pancreatic cancer, FCGR3A is linked to tumor growth, metastasis, and infiltration of M2 macrophages.

## Data Availability

All data generated or analyzed during this study are available from the corresponding author upon request.

## Appendix A

**Table A1:** Primers were used for this study

Primers	Sequence (5′-3′) forward	Sequence (5′-3′) reverse
FCGR3A	CCTCCTGTCTAGTCGGTTTGG	TCGAGCACCCTGTACCATTGA
GAPDH	TGACATCAAGAAGGTGGTGAAGCAG	GTGTCGCTGTTGAAGTCAGAGGAG

**Table A2:** siRNA sequences were used for this study

Name	Sequence
SiRNA-FCGR3A	5′-UGGAAGGACCAUAAAUUUATT-3′
